# Supplementary material for: Alveolar macrophage-expressed Plet1 is a driver of lung epithelial repair after viral pneumonia
Source: Nat Commun. 2024 Jan 2;15:87. doi: 10.1038/s41467-023-44421-6 (PMC10761876; doi:10.1038/s41467-023-44421-6)
Supplement: Supplementary file 1 — Supplementary Information [file 41467_2023_44421_MOESM1_ESM.pdf]

Supplementary Figures

Supplementary Figure 1.

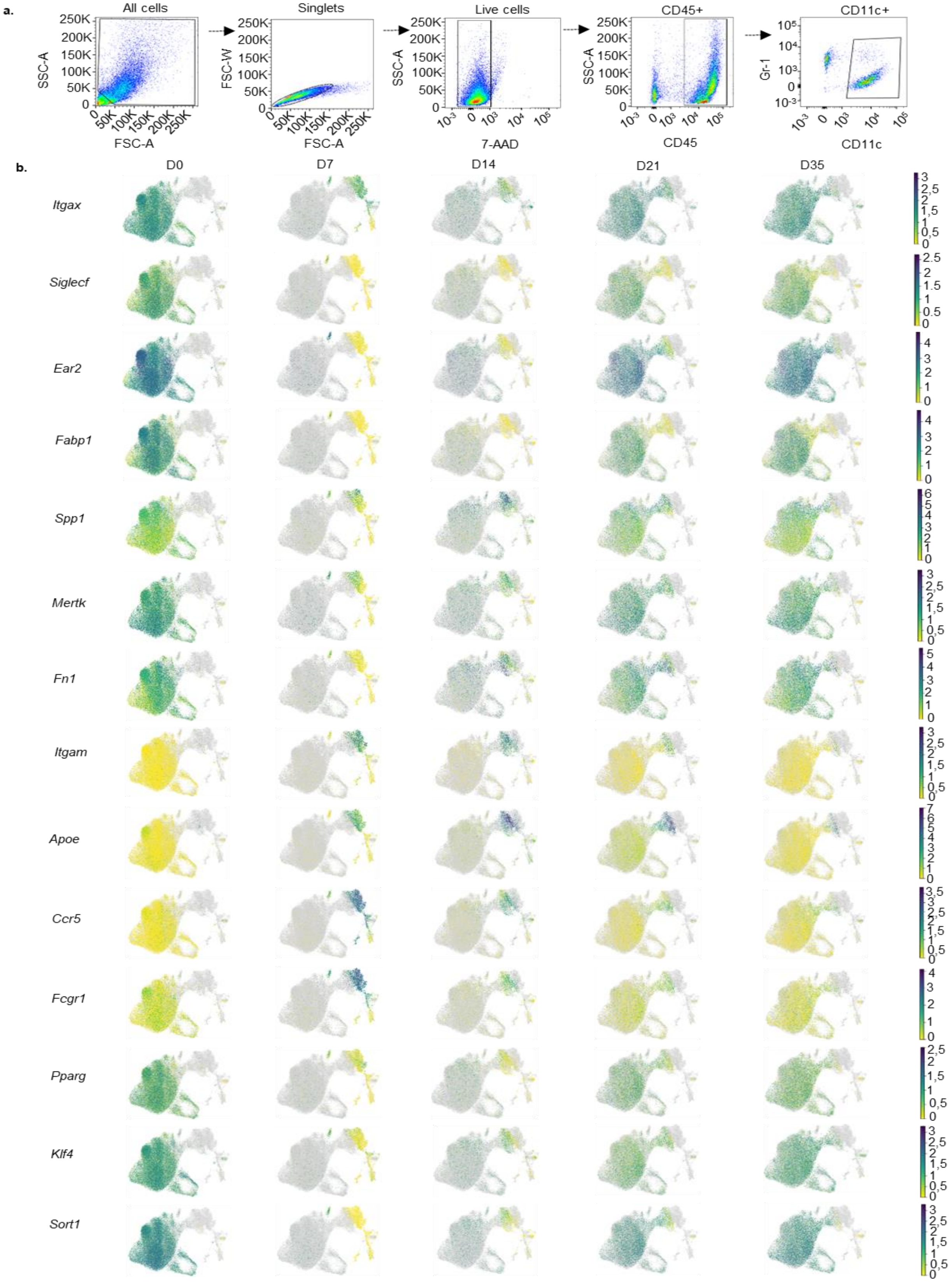

Supplementary Figure 1.

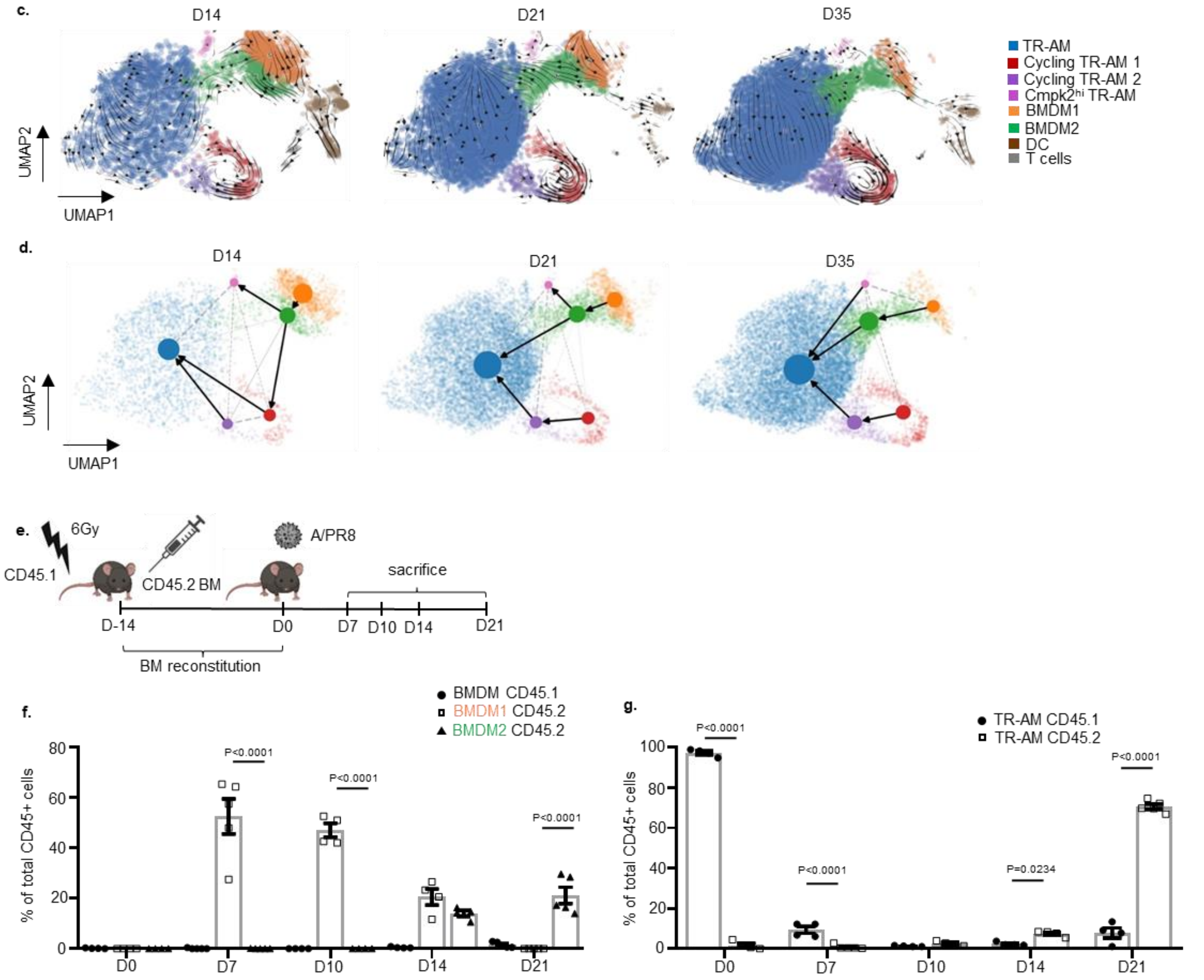

Supplementary Figure 1. a, FACS gating strategy used to flow-sort CD11c^{+} cells in BALF of IAV-infected WT mice for scRNA-seq analysis. **b**, UMAP projections of cells coloured by expression level of the selected signature genes in the TR-AM and BMDM clusters depicted in the main Fig. 1b. Yellow colour denotes minimal expression, green colour intermediate and blue colour high expression. **c**, UMAP map of the scRNA-seq experiment of Fig. 1b with embedded streamlines showing RNA velocity analysis performed with scVelo. **d**, PAGA graph reconstructing cell trajectory and connectivity between clusters shown in c. **e**, Schematic representation of the IAV infection model in CD45.1/CD45.2 BM chimeric mice. The figure was created with BioRender. **f**, Percentages of BMDM1 or BMDM2 populations present in BALF of chimeric mice before (D0 n=4) and after (D7 n=5/10 n=4/14 n=4/21 n=5) IAV infection. **g**, Percentages of CD45.1^{+} (recipient, FMo-derived) and CD45.2^{+} (donor, BMDM-derived) TR-AM in BALF of IAV-infected chimeric mice (n=4 except in CD45.2 D7 and D21 were n=5). Data are representative of three independent experiments showing mean ± SEM calculated using Two-Way ANOVA and Tukey's post-hoc test. Source data are provided as a Source Data file.

Supplementary Figure 2.

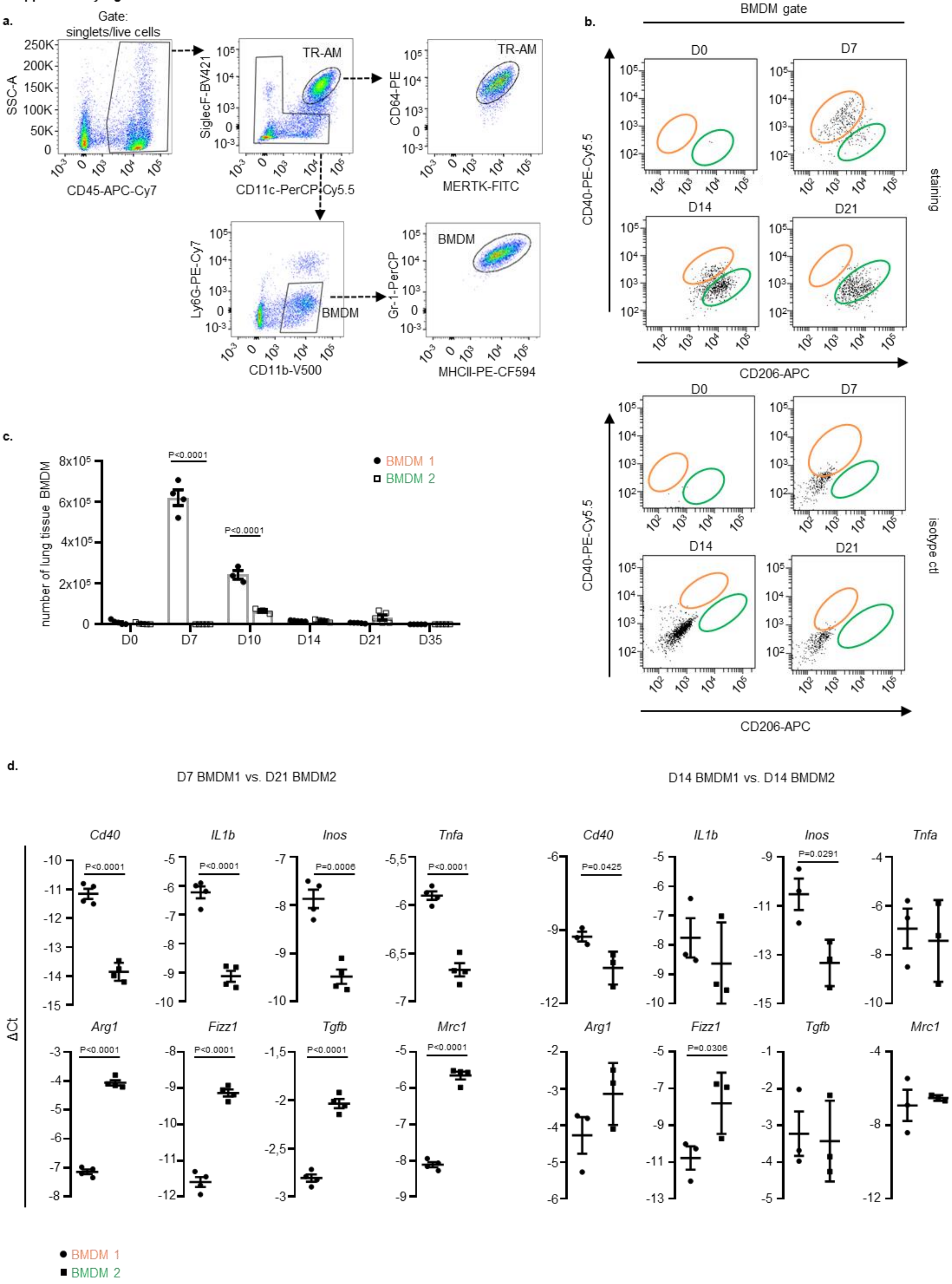

Supplementary Figure 2. Representative FACS plots of the gating strategy to identify tissue resident alveolar macrophages (**a**, TR-AM) and bone marrow-derived macrophages (**a** and **b**, BMDM) from BALF of IAV-infected mice. TR-AM were identified by the signature $CD45^{+}GR\text{-}1^{lo}Ly6G^{-}CD11b^{lo}CD11c^{hi}SiglecF^{hi}MERTK^{hi}CD64^{hi}$, BMDM1 and BMDM2 were identified by the signature $CD40^{hi}CD206^{lo}CD45^{+}Ly6C^{+}Ly6G^{-}CD11b^{hi}CD11c^{lo}SiglecF^{-}$ and $CD40^{lo}CD206^{hi}CD45^{+}Ly6C^{+}Ly6G^{-}CD11b^{hi}CD11c^{lo}SiglecF^{-}$, respectively. **b**, Representative example of dot plots for expression of CD40 (orange) and CD206 (green) in BMDM from BALF of mice over the IAV infection course (D0/7/14/21). Top row plots show staining with respective isotype controls mAbs for CD40 and CD206, bottom rows show BMDM staining with CD40 and CD206 mAbs. **c**, Quantification of BMDM1 and BMDM2 in lung tissue (obtained after BAL) of control mice (D0 n=5) or IAV infected mice at D7 (n=4)/10 (n=3)/14 (n=5)/21 (n=5)/35 (n=5) p.i.. Of note, the same FACS strategy was applied as in a, b. **d**, qPCR validation of genes expressed in BMDM1 obtained on d7 vs. BMDM2 obtained on d21 (n=4), or BMDM1 vs. BMDM2 obtained both on d14 (n=3), according to DNA microarray profiling shown in Fig. 2d. Results are expressed as ΔCt value (Ct reference-Ct target). Data are representative of four independent experiments showing mean ± SEM calculated in **c** using Two-Way ANOVA and Sidak's post-hoc test in **d** using two-sided student's t-test. Source data are provided as a Source Data file.

Supplementary Figure 3.

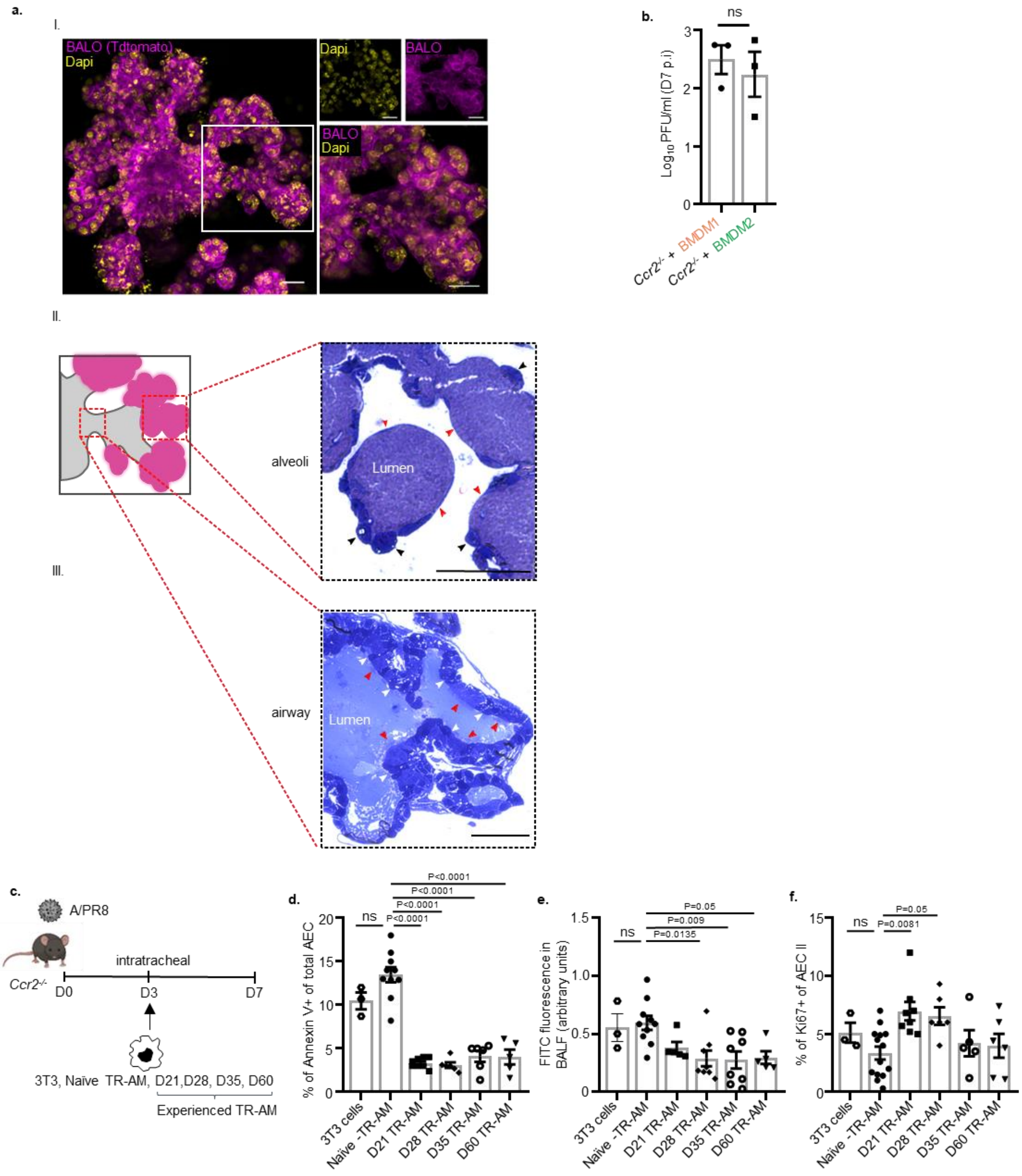

Supplementary Figure 3. a, Structural and cellular composition of murine bronchoalveolar lung organoids (BALO) generated from lung epithelial stem cells of tdTomato reporter mice[1]. (I.) depicts a representative confocal image of the BALO airway and alveolar structure (insert in left panel magnified in right panel; magenta, tdTomato+ epithelial cells; yellow, DAPI, scale bars represent 20 μm). The BALO morphology is schematically represented in the left panel of (II).

Sectioning reveals presence of alveoli (II) and airways (III). Alveoli are composed of flattened AEC I (red arrowheads) and cuboidal AEC II (black arrowheads) as shown in semi thin sections stained with Toluidine blue. Scale bar indicates 50 µm. BALO airways (III., bottom row) with secretory cells (white arrowheads) and ciliated cell (Ci, red arrowheads) in semithin sections stained with Toluidine blue (left panel). Scale bar indicates 50 µm. **b**, Virus titres quantified at D7 p.i. in the BALF of IAV infected *Ccr2*$^{-/-}$ mice, after intratracheal transfer of either BMDM1 or BMDM2 at D3 p.i.. ns = not significant, shown are means ± SEM and single data points for each group. **c**, Experimental setup of intratracheal transfer of different populations of TR-AM into IAV-infected *Ccr2*$^{-/-}$ mice (refers to data in d-f). The figure was created with BioRender. **d**, percentage of apoptotic AEC (CD31/45^{neg}EpCam^{+}Annexin V^{+}) was analysed by FACS. (3T3 n=3, Naïve-TRAM n=10, D21 TR-AM n=8, D28 TR-AM n=6, D35 TR-AM n=6, D60 TR-AM n=5) **e**, assessment of lung barrier permeability by FITC fluorescence in BALF of *Ccr2*$^{-/-}$ recipient mice after intravenous application of FITC-labeled albumin, ratios of BALF to serum fluorescence are given as arbitrary units. (3T3 n=3, Naïve-TRAM n=10, D21 TR-AM n=5, D28 TR-AM n=8, D35 TR-AM n=8, D60 TR-AM n=5) **f**, percentage of proliferating AEC II (CD31/45^{neg}EpCam^{+}T1α^{neg}Ki67^{+}) was analysed by FACS (3T3 n=3, Naïve-TRAM n=14, D21 TR-AM n=8, D28 TR-AM n=6, D35 TR-AM n=5, D60 TR-AM n=6). Bar graphs depicting single data points are representative of three independent experiments and show mean ± SEM. Significance was calculated using ANOVA followed by Tukey's post-hoc tests Source data are provided as a Source Data file.

Supplementary Figure 5.

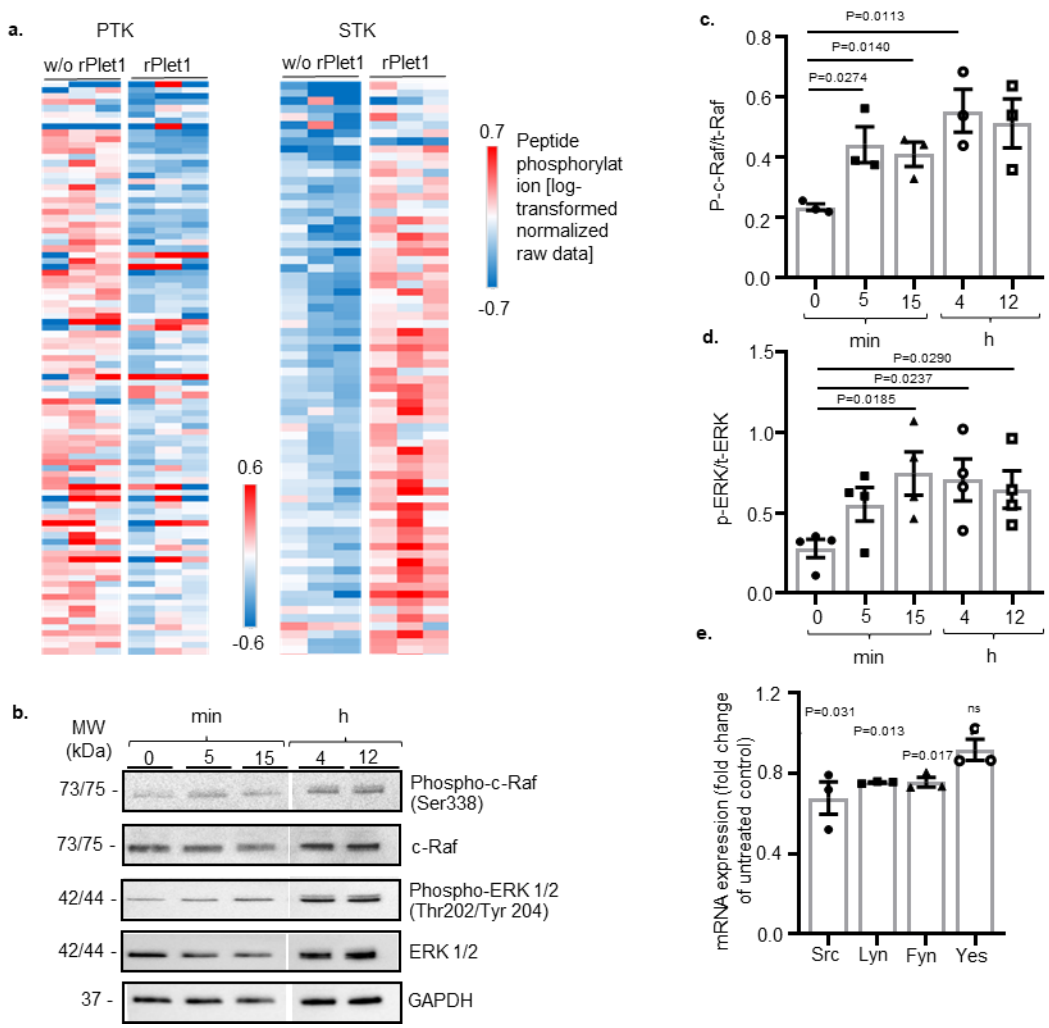

Supplementary Figure 5. a, Heatmap of log transformed mean signal intensities for PTK and STK activity profiles in murine AEC cultures treated with rPlet1 or control for 12 h. The heatmaps visualize the major differences in predicted phosphorylation intensity among cells treated with rPlet1, where blue denotes low activity and red denotes high activity of kinases. **b**, Representative Western blot images showing protein levels of total and phosphorylated c-Raf, ERK, and GAPDH, in lysates of primary murine AEC treated with rPlet1 for 0 min (control), 5 min, 15 min, 4 h and 12 h. Note that all band images have been cut between the 15 min and 4 h lanes. **c-d**, Bar graphs represent ratio of phosphorylated protein over total protein normalized by protein loading control GAPDH for the indicated kinase. (c-Raf n=3; ERK n=4) **e**, mRNA expression of Src family genes (*Src*, *Yes*, *Fyn*, *Lyn)* in AEC treated with rPlet1 for 2 h. Results are expressed as fold change over untreated control (n=3). Data are representative of three independent experiments; bar graphs show means ± SEM and single data points. Statistical difference between control and treated samples was evaluated using two-sided Student's test. Source data are provided as a Source Data file.

Supplementary Figure 6.

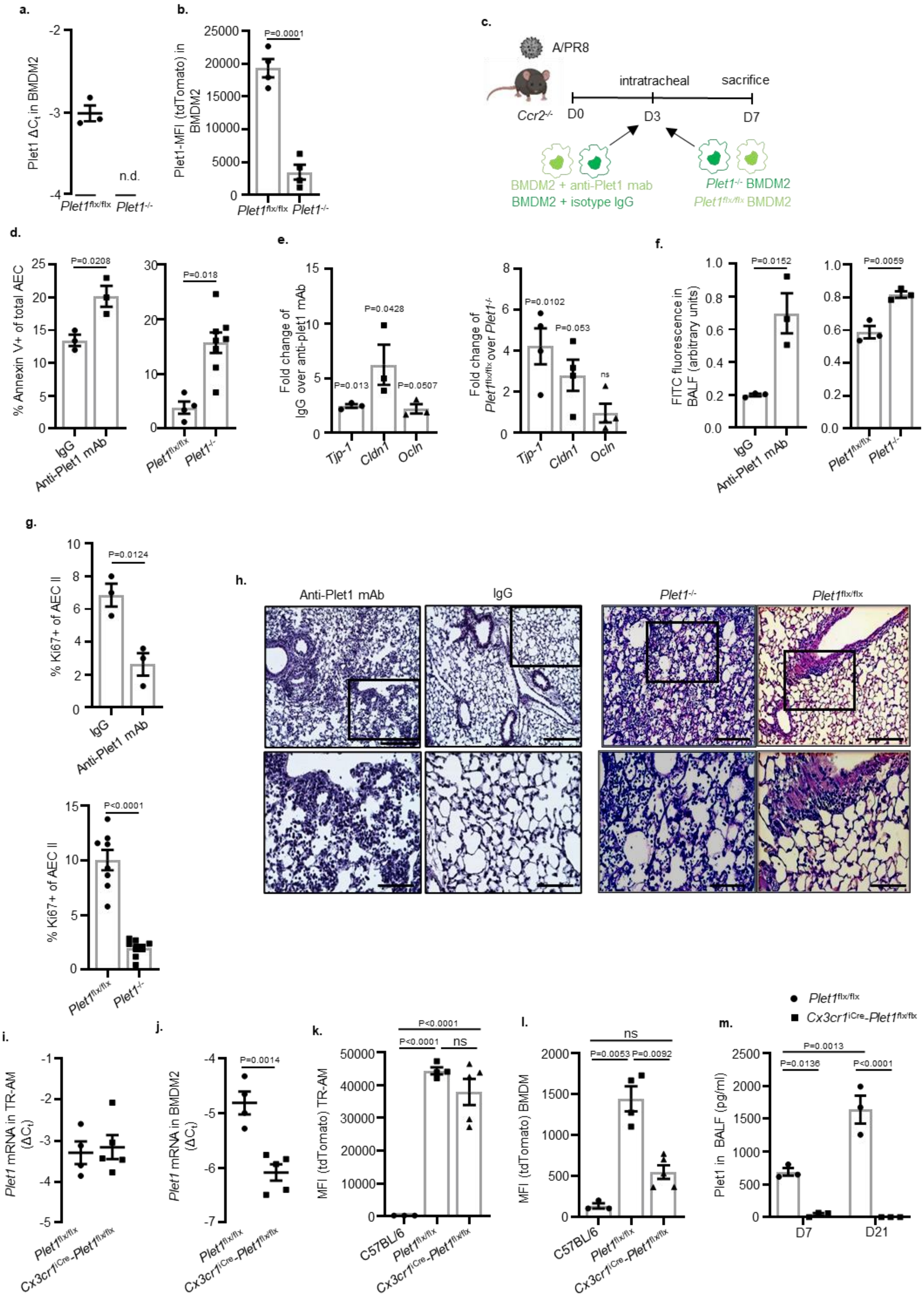

Supplementary Figure 6.

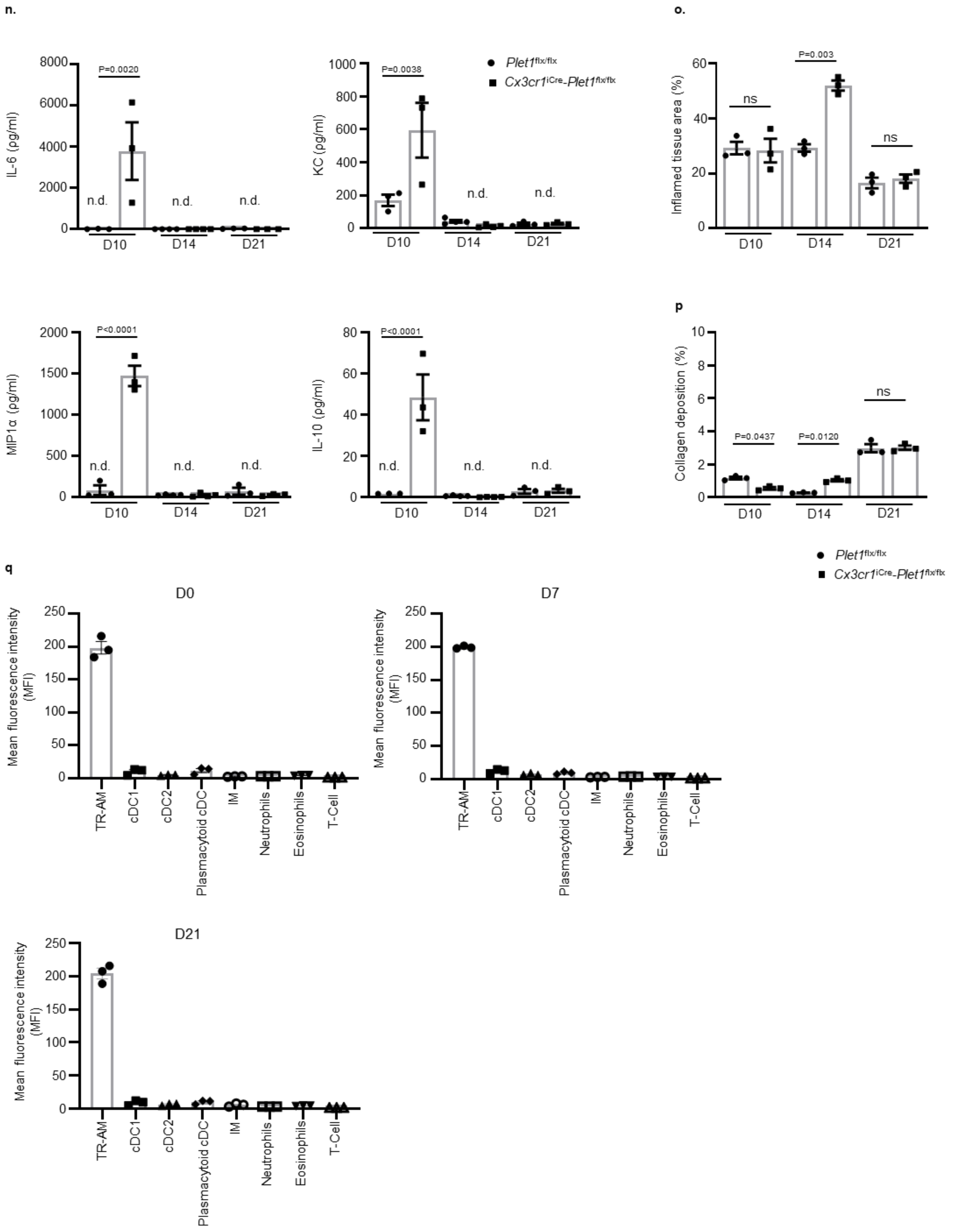

Supplementary Figure 6. a, *Plet1* mRNA expression in BMDM2 flow-sorted from BALF of control *Plet1*$^{tdtomato\text{-}flox/flox}$ mice (*Plet1*$^{flx/flx}$), and global *Plet1* KO mice (*Plet1*$^{-/-}$) by qPCR. n.d = not detected. (n=3) **b**, FACS analysis of MFI (mean fluorescence intensity) of tdTomato (Plet1-reporter) expression in BMDM2 from BALF of *Plet1*$^{flx/flx}$ and *Plet1*$^{-/-}$ mice obtained by FACS sorting. (n=3) **c**, Schematic depiction of experiments analysed in d-h**. d**, Annexin V^{+} percentage of AEC. (For IgG treatment n=3; *Plet1*$^{flx/flx}$ mice n=4, *Plet1*$^{-/-}$ mice n=8) **e**, mRNA expression (qPCR) of tight junction proteins in the lung. (n=3 for IgG treatment, n=4 in *Plet1*$^{flx/flx}$ and *Plet1*$^{-/-}$ mice) **f**, Quantification of barrier dysfunction by FITC-Albumin fluorescence analysis in BALF. (n=3) **g**, Ki67^{+} percentage of AEC II indicating proliferation. (n=3 for IgG treatment, n=8 in *Plet1*$^{flx/flx}$ and *Plet1*$^{-/-}$ mice) **h**, Representative images of lung tissue sections (stained with H&E). Bottom images are magnification of the top images (black squares); scale bars represent 100 µm. **i**, *Plet1* mRNA expression in TR-AM from Txf-treated *Plet1*$^{flx/flx}$ (n=4) and *Cx3cr1*^{iCre}*Plet1*$^{-/-}$ (n=5) mice (D0) and **j**, in BMDM2 from *Plet1*$^{flx/flx}$ (n=4) and *Cx3cr1*^{iCre}*Plet1*$^{-/-}$ (n=5) mice (D21 p.i.). **k**, tdTomato-MFI (Plet1 reporter gene) in TR-AM from Txf-treated *Plet1*$^{flx/flx}$ (n=4) and *Cx3cr1*^{iCre}*Plet1*$^{-/-}$ (n=5) mice or C57BL/6 WT mice (n=3) (D0) and **l**, in BMDM2 from *Plet1*$^{flx/flx}$ (n=4) and *Cx3cr1*^{iCre}*Plet1*$^{-/-}$ (n=5) mice or C57BL/6 WT mice (n=3) (D21 p.i.). **m**, Quantification of soluble Plet1 in BALF of IAV-infected, Txf-treated *Plet1*$^{flx/flx}$ or *Cx3cr1*^{iCre}-*Plet1*$^{flx/flx}$ mice at D7/21 p.i.. (n=3) **n**, Cytokine quantification in BALF of *Plet1*$^{flx/flx}$ and *Cx3cr1*^{iCre}-*Plet1*$^{flx/flx}$ mice on days 10 (n=3), 14 (n=4) and 21 (n=3) after IAV infection. Percentage of **o**, inflamed tissue area, and **p**, collagen-positive lung tissue areas after staining with Mason´s trichrome in *Plet1*$^{flx/flx}$ and *Cx3cr1*^{iCre}-*Plet1*$^{flx/flx}$ mice on days 10, 14 and 21 after IAV infection (n=3). **q**, Expression of Plet1 in CD45+ cells of lung tissue on days 0, 7 and 21 post IAV infection. The results were obtained by flow cytometry analysis on Plet1 reporter mice and expressed as mean fluorescence intensity (n=3). Gating and definition of leukocyte populations were performed according to the gating strategy published by Misharin et al[2]. Data are representative of three independent experiments; bar graphs show means ± SEM and single data points. ns = not significant. Significance was calculated using two-sided student's t-test on **a-j,** ANOVA followed by Tukey's post-hoc tests on **k-p,** except on **l** where Brown Forsythe and Welch ANOVA followed by Games-Howell's test was applied. Source data are provided as a Source Data file.

Supplementary Figure 7.

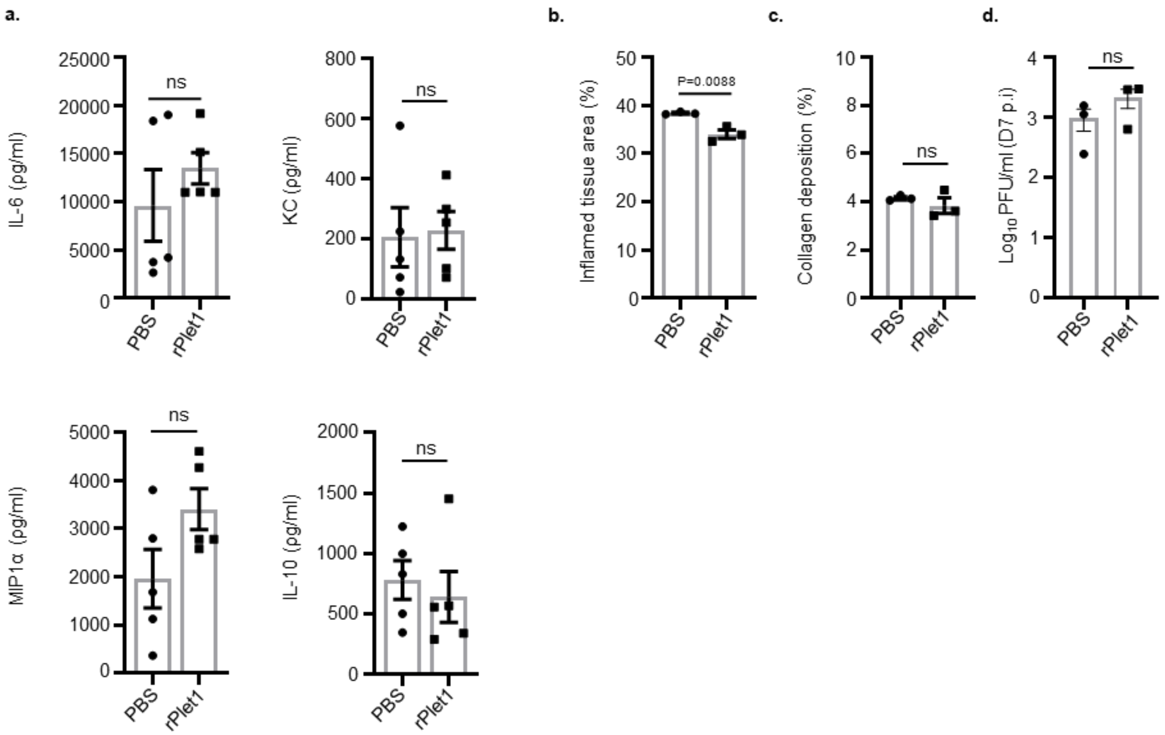

Supplementary Figure 7. a, Cytokine quantification in BALF of IAV infected mice treated with PBS or rPlet1 (5 µg) (n=5). Percentage of **b** (n=3)**,** inflamed tissue area and **c** (n=3)**,** collagen-positive lung tissue areas after staining with Mason´s trichrome of IAV infected mice treated with PBS or rPlet1 (5 µg). **d**, Quantification of virus titres in BALF of IAV infected mice treated with PBS or rPlet1 (5 µg) (n=3). Data are representative of three independent experiments; bar graphs show means ± SEM ns = not significant. Significance was calculated using two-sided student's t-test. Source data are provided as a Source Data file.

Supplementary Table. Patient characteristics of the two cohorts analysed for PLET1 and total protein concentrations in BALF (Fig. 7a-c).

Cohort	BAL indication/diagnosis	Male Gender	Age (y)	Invasi ventila
Control (non-infectious/ non-inflammatory lung disease)	congenital cystic adenomatoid malformation	yes	16	no
	interstitial lung disease (suspected)	no	40	no
	sarcoidosis (suspected)	yes	24	no
	chronic cough	yes	45	no
	post lung transplant	yes	55	no
	non small cell lung cancer	yes	66	no
	post lung transplant	yes	45	no
	post lung transplant	yes	66	no
	post lung transplant	no	63	no
Influenza Virus-induced lung injury	Influenza (PCR-confirmed), ARDS (Berlin definition)	yes	62	yes
	Influenza (PCR-confirmed), ARDS (Berlin definition)	yes	29	yes
	Influenza (PCR-confirmed), ARDS (Berlin definition)	yes	65	yes
	Influenza (PCR-confirmed), ARDS (Berlin definition)	yes	69	yes
	Influenza (PCR-confirmed), ARDS (Berlin definition)	yes	46	yes
	Influenza (PCR-confirmed), ARDS (Berlin definition)	no	51	yes
	Influenza (PCR-confirmed), ARDS (Berlin definition)	yes	50	yes
	Influenza (PCR-confirmed), ARDS (Berlin definition)	yes	53	yes
	Influenza (PCR-confirmed), ARDS (Berlin definition)	no	40	yes
	Influenza (PCR-confirmed), ARDS (Berlin definition)	yes	64	yes
	Influenza (PCR-confirmed), ARDS (Berlin definition)	yes	63	yes
	Influenza (PCR-confirmed), ARDS (Berlin definition)	no	38	yes
	Influenza (PCR-confirmed), ARDS (Berlin definition)	yes	43	yes
	Influenza (PCR-confirmed), ARDS (Berlin definition)	yes	42	yes

References

1. Vazquez-Armendariz AI, Heiner M, El Agha E, Salwig I, Hoek A, Hessler MC, *et al.* Multilineage murine stem cells generate complex organoids to model distal lung development and disease. *Embo j* 2020, **39**(21)**:** e103476.

2. Misharin AV, Morales-Nebreda L, Mutlu GM, Budinger GR, Perlman H. Flow cytometric analysis of macrophages and dendritic cell subsets in the mouse lung. *Am J Respir Cell Mol Biol* 2013, **49**(4)**:** 503-510.
