# Supplementary material for: Alveolar macrophage-expressed Plet1 is a driver of lung epithelial repair after viral pneumonia
Source: Nat Commun. 2024 Jan 2;15:87. doi: 10.1038/s41467-023-44421-6 (PMC10761876; doi:10.1038/s41467-023-44421-6)
Supplement: Supplementary file 3 — Description of Additional Supplementary Files [file 41467_2023_44421_MOESM3_ESM.pdf]

Description of Additional Supplementary Files

File Name: Supplementary Data 1
Description: Antibody List

File Name: Supplementary Data 2
Description: Primer List
