# Supplementary material for: Alveolar macrophage-expressed Plet1 is a driver of lung epithelial repair after viral pneumonia
Source: Nat Commun. 2024 Jan 2;15:87. doi: 10.1038/s41467-023-44421-6 (PMC10761876; doi:10.1038/s41467-023-44421-6)
Supplement: Supplementary file 6 — Reporting Summary [file 41467_2023_44421_MOESM6_ESM.pdf]

nature portfolio

Corresponding author(s): Susanne Herold

Last updated by author(s): Nov 27, 2023

Reporting Summary

Nature Portfolio wishes to improve the reproducibility of the work that we publish. This form provides structure for consistency and transparency in reporting. For further information on Nature Portfolio policies, see our Editorial Policies and the Editorial Policy Checklist.

Statistics

For all statistical analyses, confirm that the following items are present in the figure legend, table legend, main text, or Methods section.

n/a	Confirmed	
☐	☒	The exact sample size (*n*) for each experimental group/condition, given as a discrete number and unit of measurement
☐	☒	A statement on whether measurements were taken from distinct samples or whether the same sample was measured repeatedly
☐	☒	The statistical test(s) used AND whether they are one- or two-sided<br>*Only common tests should be described solely by name; describe more complex techniques in the Methods section.*
☒	☐	A description of all covariates tested
☐	☒	A description of any assumptions or corrections, such as tests of normality and adjustment for multiple comparisons
☐	☒	A full description of the statistical parameters including central tendency (e.g. means) or other basic estimates (e.g. regression coefficient) AND variation (e.g. standard deviation) or associated estimates of uncertainty (e.g. confidence intervals)
☐	☒	For null hypothesis testing, the test statistic (e.g. *F*, *t*, *r*) with confidence intervals, effect sizes, degrees of freedom and *P* value noted<br>*Give P values as exact values whenever suitable.*
☒	☐	For Bayesian analysis, information on the choice of priors and Markov chain Monte Carlo settings
☒	☐	For hierarchical and complex designs, identification of the appropriate level for tests and full reporting of outcomes
☒	☐	Estimates of effect sizes (e.g. Cohen's *d*, Pearson's *r*), indicating how they were calculated

Our web collection on statistics for biologists contains articles on many of the points above.

Software and code

Policy information about availability of computer code

Data collection	FLX 800, Bio-Tek instruments, AB StepOnePlus Detection System (Applied Bioscience), BD LSRFortessa flow cytometer and BD AriaIII (BD Biosciences), EVOS FL imaging system (Thermo Fisher Scientific), Epson Perfection V500 Photo scan (Epson), iMarkTM, Bio-Rad, Millicell-ERS2 device, PamStation®12 platform (Pamgene, BJ´s-Hertogenbosch, The Netherlands), western blotting system (GE healthcare, München, Germany), BioPlex MAGPIX Multiplex Reader (BIO-RAD, United States), Leica SP8 confocal microscope (LAS X 3.5.7 software), SP5 (software version LAS AF 2.7.3), InnoScan 900 instrument, Illuminas bcl2fastq (2.19.0.316).
Data analysis	FACS Diva (v7.0) and FlowJo (v10.7.0), ImageJ (v1.53K), Mapix (v6.5.0), clusterProfiler, fgsea packages in R (R Core Team, 2021), limma package from BioConductor, Scanpy (v1.7.2), Python (v3.8.10,) scVelo, Image Lab (v6.1), GraphPad Prism (v9.3.1), Bio-Plex Data Pro software, R (v3.6.1) (R Core Team. R, URL https://www.R-project.org/ 2015), scMCA R package (https://github.com/ggjlab/scMCA), custom made macro (https://doi.org/10.5281/zenodo.10135192) to perform pixel classification using a pre-trained Ilastik model. Code for the scRNA-seq analysis is accessible at https://github.com/agbartkuhn/Pervizaj-Oruqaj_Plet1_sc_analysis.

For manuscripts utilizing custom algorithms or software that are central to the research but not yet described in published literature, software must be made available to editors and reviewers. We strongly encourage code deposition in a community repository (e.g. GitHub). See the Nature Portfolio guidelines for submitting code & software for further information.

Data

Policy information about availability of data

All manuscripts must include a data availability statement. This statement should provide the following information, where applicable:

- Accession codes, unique identifiers, or web links for publicly available datasets
- A description of any restrictions on data availability
- For clinical datasets or third party data, please ensure that the statement adheres to our policy

The microarray data used in this publication have been deposited in NCBI's Gene Expression Omnibus and are accessible through GEO Series accession number GSE208000 (https://www.ncbi.nlm.nih.gov/geo/query/acc.cgi?acc=GSE208000). The scRNA-seq data have been deposited in NCBI's Gene Expression Omnibus and are accessible through GEO Series accession number GSE208294. For the analysis of fibrosis of lung sections we used a custom made macro (https://doi.org/10.5281/zenodo.10135192) to perform pixel classification using a pre-trained Ilastik model. Code for the scRNA-seq analysis is accessible at https://github.com/agbartkuhn/Pervizaj-Oruqaj_Plet1_sc_analysis.

Human research participants

Policy information about studies involving human research participants and Sex and Gender in Research.

Reporting on sex and gender	Biological sex is given in the information. We did not stratify our analyses on sex/gender.
Population characteristics	BALF samples were from patients with confirmed influenza A virus (IAV) -induced ARDS, verified by PCR analysis. ARDS was diagnosed according to the Berlin definition. Controls were defined as given below.<br>Further characteristics are given in the respective table 1 (BALF samples) as a supplementary material.
Recruitment	The inclusion criteria for patients with ARDS in our study was a diagnosis of pneumonia-associated ARDS, meeting the Berlin ARDS definition, that received BAL procedure for diagnostic or trial-related reasons. The inclusion of control patients was based on patients after written consent that received BAL procedure due to diagnostic reasons, but had no deviations compared to standard values of cellularity in healthy patients (subpopulation composition; cell numbers), and no diagnosis of pneumonia and/or ARDS .
Ethics oversight	Human lung tissue and BALF samples were obtained from patients who underwent lobectomy or received bronchoscopy, respectively, for diagnostic reasons. Use of human lung tissue and BALF samples was approved by the University of Giessen Ethics Committee (Nr 85-93 and 58-15).

Note that full information on the approval of the study protocol must also be provided in the manuscript.

Field-specific reporting

Please select the one below that is the best fit for your research. If you are not sure, read the appropriate sections before making your selection.

☒ Life sciences ☐ Behavioural & social sciences ☐ Ecological, evolutionary & environmental sciences

For a reference copy of the document with all sections, see nature.com/documents/nr-reporting-summary-flat.pdf

Life sciences study design

All studies must disclose on these points even when the disclosure is negative.

Sample size	The number of animals used was limited to the detection of significant statistical differences between experimental groups, estimated according to previously published results. Determination of sample size was in accordance with the 3R principle in line with the regulation of the animal ethical committee of the State of Hesse (Regierungspräsidium Giessen) and by the Institutional Ethics Committee at the IBioBA Institute
Data exclusions	During the experiments conducted, no data exclusion was implemented.
Replication	Experiment replication was enough to ensure data reproducibility. All experiments were performed at least two times and most of them were performed three times.
Randomization	Mice between 10 and 14 weeks of age were randomly assigned to each experimental group together with their respective littermate controls. In experiments (also in those not explicitly detailed here), sample allocation into experimental groups was conducted through a randomization process. Random allocation was employed to ensure a well-balanced representation of participants (animals) across the different experimental groups, minimizing potential biases and confounding factors. This approach enhances the internal validity of our study, as it promotes equal distribution of both known and unknown variables among the groups. The random allocation method was chosen to strengthen the robustness and generalizability of our findings.
Blinding	Except from bioinformatic data that were performed by bioinformaticians with no knowledge of experimental conditions, the rest of the

Blinding

analysis were not blinded.
In experiments outside the realm of bioinformatics data, blinding was not implemented due to the inherent nature of the study design. The specific characteristics of the experiments, such as the type of interventions and the outcome measurements, made blinding impractical or unfeasible. The decision was made considering the unique requirements of our study protocol, aiming to maintain transparency and rigor in our experimental procedures.

Reporting for specific materials, systems and methods

We require information from authors about some types of materials, experimental systems and methods used in many studies. Here, indicate whether each material, system or method listed is relevant to your study. If you are not sure if a list item applies to your research, read the appropriate section before selecting a response.

Materials & experimental systems

n/a	Involved in the study	
☐	☒	Antibodies
☒	☐	Eukaryotic cell lines
☒	☐	Palaeontology and archaeology
☐	☒	Animals and other organisms
☒	☐	Clinical data
☒	☐	Dual use research of concern

Methods

n/a	Involved in the study	
☒	☐	ChIP-seq
☐	☒	Flow cytometry
☒	☐	MRI-based neuroimaging

Antibodies

Antibodies used

The fluorochrome-labeled antibodies used for FACS analysis are:
CD326 (Ep-CAM) APC-Cy7, (clone G8.8), (1/100 dilution), (Cat No 118218), Biolegend
CD24 PE-Cy7, (clone M1/69) , (1/100 dilution), (Cat No 101821), Biolegend
CD31 Alexa fluor 488, (clone MEC13.3), (1/50 dilution), (Cat No 102513), Biolegend
CD31 Pacific blue, (clone 390), (1/50 dilution), (Cat No 102413), Biolegend
CD45 FITC, (clone 30-F11) ,(1/100 dilution), (Cat No 103107), Biolegend
CD45 APC-Cy7, (clone 30-F11) ,(1/100 dilution), (Cat No 103115), Biolegend
CD45 Pacific blue, (clone 30-F11), (1/100 dilution), (Cat No 103125), Biolegend
T1α/podoplanin APC, (clone 8.1.1), (1/20 dilution), (Cat No 127409), Biolegend
Ki67 FITC, (clone 16A8), (1/50 dilution), (Cat No 652409), Biolegend
Ki67 BV605, (clone 16A8), (1/50 dilution), (Cat No 652413), Biolegend
Ki67 PE, (clone 16A8), (1/50 dilution), (Cat No 652403), Biolegend
FITC Rat IgG2a, κ Isotype Ctrl Antibody (clone RTK2758), (1/50), (Cat No 400505), Biolegend
Brilliant Violet 605™ Rat IgG2a, κ Isotype Ctrl Antibody (clone RTK2758), (1/50 dilution), (Cat No 400539), Biolegend
PE Rat IgG2a, κ Isotype Ctrl Antibody (clone RTK2758), (1/50 dilution), (Cat No 400508), Biolegend
Annexin V Alexa fluor 647, (clone 640907), (1/20 dilution), (Cat No A23204), Invitrogen
Annexin V PE, (clone 640907), (1/20 dilution), (Cat No A35111), Invitrogen
GR-1 PE-Cy7, (clone RB6-8C5) ,(1/100 dilution), (Cat No 108415), Biolegend
GR-1 PerCP, (clone RB6-8C5), (1/100 dilution), (Cat No 108425), Biolegend
Ly6G PE-Cy7, (clone 1A8), (1/50) dilution), (Cat No 127617), Biolegend
Ly6G APC , (clone 1A8), (1/50 dilution), (Cat No 127613), Biolegend
Ly6C FITC, (clone AL-21), (1/20 dilution), (Cat No 553104), BD Biosciences
Siglec-F PE, (clone E50-2440), (1/50 dilution), (Cat No 562068), BD Biosciences
Siglec-F BV421, (clone E50-2440), (1/50) dilution), (Cat No 562681), BD Biosciences
CD11c Percp.Cy5.5, (clone N418), (1/20 dilution), (Cat no 117327), Biolegend
CD11b V500, (clone M1/70), (1/50 dilution), (Cat No 562127), BD Biosciences
CD11b BV421, (clone M1/70), (1/50 dilution), (Cat No 562605), BD Biosciences
MERTK FITC, (clone 2B10C42), (1/50 dilution), (Cat No 151503), Biolegend
CD64 PE, (clone X54-5/7.1), (1/20 dilution), (Cat No 139303), Biolegend
MHCII FITC, (clone AF6-120.1), (1/50 dilution), (Cat No 562011), BD Biosciences
MHCII PE-CF594, (clone AF6-120.1), (1/50 dilution), (Cat No 562824), BD Biosciences
CD45.1 FITC, (clone A20), (1/50 dilution), (Cat No 110705), Biolegend
CD45.2 APC-Cy7, (clone 104), (1/50 dilution), (Cat No 109823), Biolegend
CD206 APC, (clone C068C2), (1/20 dilution), (Cat No 141707), Biolegend
Rat IgG2a, κ Isotype Ctrl Antibody APC, (clone RTK2758), (1/20 dilution), (Cat No 400511), Biolegend
CD40 Pe-Cy5, (clone 3/23), (1/20 dilution), (Cat No 124617), Biolegend
Rat IgG2a, κ Isotype Ctrl Antibody PE-Cy5, (clone RTK2758), (1/20 dilution), (Cat No 400509), Biolegend
7-AAD (1/100 dilution), (Cat No 420403), Biolegend
Sytox (1/1000 dilution), (Cat No S34862), Thermofisher Scientific

For AECs isolation the antibodies below were used:
Biotin Rat Anti-Mouse CD31, (clone MEC13.3), (Cat No 553371), BD Biosciences
Biotin Rat Anti-Mouse CD16/CD32, (clone 2.4G2) (Cat NO 553143), BD Biosciences
Biotin Rat Anti-Mouse CD45, (clone 30-F11), (Cat No 553078), BD Biosciences

For Western blot the following primary antibodies were used:
c-Raf Antibody, (1:1,000 dilution), (Cat No 9422), Cell Signaling Technology
Phospho-c-Raf (Ser338) (56A6) Rabbit mAb, (1:1,000 dilution), (Cat No 9427), Cell Signaling Technology
p44/42 MAPK (Erk1/2) (137F5) Rabbit mAb, (1:1,000 dilution), (Cat No 4695), Cell Signaling Technology
Phospho-p44/42 MAPK (Erk1/2) (Thr202/Tyr204) (D13.14.4E) XP® Rabbit mAb, (1:1,000 dilution), (Cat No 4370), Cell Signaling Technology
GAPDH (14C10) Rabbit mAb, (1:2,000 dilution), (Cat No 2118), Cell Signaling Technology
Anti-rabbit IgG, HRP-linked Antibody, (1:2,000 dilution), (Cat No 7074S),Cell Signaling Technology

Mouse PLET-1 Antibody, (Cat No MAB6917), (Clone 700738), R&D Systems

Validation

#CD326 (Ep-CAM) APC-Cy7, (clone G8.8), (Cat No 118218), Biolegend. The antibody has been validated for reactivity with mouse tissue or proteins. It is optimized for use in flow cytometry. According to manufacturer's website the recommended usage for flow cytometric staining, the suggested use of this reagent is ≤ 0.25 µg per 106 cells in 100 µl volume. Product Citations:
1.Ochiai S, et al. 2014. J Immunol. 193:2504. PubMed
2.Vercauteren Drubbel A, et al. 2021. Cell Stem Cell. . PubMed
3.Zwarycz B, et al. 2018. Cell Mol Gastroenterol Hepatol. 7:1. PubMed
Antibody profile in Antibody registry: https://www.antibodyregistry.org/AB_2098648

CD24 PE-Cy7, (clone M1/69), (Cat No 101821), Biolegend. The antibody has been validated for reactivity with mouse tissue or proteins. It is optimized for use in flow cytometry. According to manufacturer's website the recommended usage for flow cytometric staining, the suggested use of this reagent is ≤ 0.25 µg per 106 cells in 100 µl volume. Product Citations:
1. Song W, et al. 2019. Cell Res. 29:206. PubMed
2. Kim S, et al. 2020. Immunity. 53(4):759-774.e9. PubMed
3. Schneider C, et al. 2018. Cell. 174:271. PubMed
Antibody profile in Antibody registry: https://www.antibodyregistry.org/AB_756047

CD31 Alexa fluor 488, (clone MEC13.3), (Cat No 102513), Biolegend. The antibody has been validated for reactivity with mouse tissue or proteins. It is optimized for use in flow cytometric analysis of antibody surface-stained cells. According to manufacturer's website the suggested use of this reagent is ≤ 2.0 µg per million cells in 100 µl volume. Product Citations:
1.Wang C, et al. 2021. Cell Rep. 37:110021. PubMed
2.Gui J, et al. 2020. Nat Cancer. 1:603. PubMed
3.Salei N, et al. 2020. J Am Soc Nephrol. 31:257. PubMed
Antibody profile in Antibody registry: https://www.antibodyregistry.org/AB_493413

CD45 FITC, (clone 30-F11), (Cat No 103107), Biolegend. The antibody has been validated for reactivity with mouse tissue or proteins. It is optimized for use in flow cytometric analysis of antibody surface-stained cells. According to manufacturer's website the suggested use of this reagent is ≤ 0.25 µg per 106 cells in 100 µl volume. Product Citations:
1.Garo LP, et al. 2021. Nat Commun. 12:2419. PubMed
2.Hutton C, et al. 2021. Cancer Cell. 39:1227. PubMed
3.Nederlof R, et al. 2022. Front Immunol. 13:908023. PubMed
4.Kataru RP, et al. 2021. Sci Signal. 14:. PubMed
Antibody profile in Antibody registry: https://www.antibodyregistry.org/AB_312972

CD45 APC-Cy7, (clone 30-F11), (Cat No 103115), Biolegend. The antibody has been validated for reactivity with mouse tissue or proteins. It is optimized for use in flow cytometric analysis of antibody surface-stained cells. According to manufacturer's website the suggested use of this reagent is = 0.25 µg per 106 cells in 100 µl volume.. Product Citations:
1.Heindl S, et al. 2021. J Exp Med. 218:. PubMed
2.Stefkovich M, et al. 2021. Mol Metab. 54:101357. PubMed
3.Wang Q, et al. 2020. Radiat Environ Biophys. 59:89. PubMed
4.Kim MY, et al. 2021. JCI Insight. 6:. PubMed
Antibody profile in Antibody registry: https://www.antibodyregistry.org/AB_312980

#CD45 Pacific blue, (clone 30-F11), (Cat No 103125), Biolegend. The antibody has been validated for reactivity with mouse tissue or proteins. It is optimized for use in flow cytometric analysis of antibody surface-stained cells. According to manufacturer's website the suggested use of this reagent is ≤ 0.25 µg per 106 cells in 100 µl volume. Product Citations:
1.Aguilar EG, et al. 2021. Blood Adv. 5:4219. PubMed
2.Marchelletta RR, et al. 2021. J Clin Invest. 131:. PubMed
3.Quintero H, et al. 2022. Cell Rep. 40:111324. PubMed
Antibody profile in Antibody registry: https://www.antibodyregistry.org/AB_493536

#T1α/podoplanin APC, (clone 8.1.1), (Cat No 127409), Biolegend. The antibody has been validated for reactivity with mouse tissue or proteins. It is optimized for use in flow cytometric analysis of antibody surface-stained cells. According to manufacturer's website the suggested use of this reagent ≤0.25 µg per million cells in 100 µl volume. Product Citations:
1.Hutton C, et al. 2021. Cancer Cell. 39:1227. PubMed
2.Buechler MB, et al. 2021. Nature. 593:575. PubMed
3.Urban SK, et al. 2020. Liver Int. 40:3103. PubMed
Antibody profile in Antibody registry: https://www.antibodyregistry.org/AB_10612940

#Ki67 FITC, (clone 16A8), (Cat No 652409), Biolegend. The antibody has been validated for reactivity with mouse tissue or proteins. It is optimized for use in flow cytometric analysis of antibody surface-stained cells. According to manufacturer's website the suggested use of this ≤0.5 µg per million cells in 100 µl volume. Product Citations:

1.Lawson H, et al. 2021. Stem Cell Reports. 16:2784. PubMed
2.Hu XF, et al. 2021. Theranostics. 3796:11. PubMed
3.Bhattacharjee A, et al. 2021. Immunity. 54(8):1745-1757.e7. PubMed
Antibody profile in Antibody registry: https://www.antibodyregistry.org/AB_2562140

Ki67 BV605, (clone 16A8), (Cat No 652413), Biolegend. The antibody has been validated for reactivity with mouse tissue or proteins. It is optimized for use in flow cytometric analysis of antibody surface-stained cells. According to manufacturer's website the suggested use of this ≤0.25 µg per million cells in 100 µl volume. Product Citations:
1.Best SA, et al. 2018. Cell Metab. 27:935. PubMed
2.Dumas AA, et al. 2020. EMBO J. 39:e103790. PubMed
3.Rivadeneira DB, et al. 2020. Immunity. 51(3):548-560. PubMed
Antibody profile in Antibody registry: https://www.antibodyregistry.org/AB_2562664

Ki67 PE, (clone 16A8), (Cat No 652403), Biolegend. The antibody has been validated for reactivity with mouse tissue or proteins. It is optimized for use in flow cytometric analysis of antibody surface-stained cells. According to manufacturer's website the suggested use of this ≤0.5 µg per million cells in 100 µl volume. Product Citations:
1. Wu M, et al. 2021. Nat Commun. 12:3500. PubMed
2.Liang J, et al. 2016. Nat Med. 22:1285-1293. PubMed
3.Sizova O, et al. 2018. Mol Cancer Res. 16:1652. PubMed
Antibody profile in Antibody registry: https://www.antibodyregistry.org/AB_2561524

#FITC Rat IgG2a, κ Isotype Ctrl Antibody (clone RTK2758), (Cat No 400505), Biolegend. It is optimized for use in flow cytometric analysis as an isotype control after screening on a variety of resting, activated, live, and fixed mouse, rat and human tissues. According to manufacturer's website each lot of this antibody is quality control tested by immunofluorescent staining with flow cytometric analysis as negative control. Use at concentrations comparable to those of the specific antibody of interest. Product citations:
1.Hattori Y, et al. 2022. J Neurosci. 42:362. PubMed
2.Reglero-Real N, et al. 2021. Immunity. :. PubMed
3.Miao L, et al. 2020. Theranostics. 0.7625. PubMed
Antibody profile in Antibody registry: https://www.antibodyregistry.org/AB_2736919

Brilliant Violet 605™ Rat IgG2a, κ Isotype Ctrl Antibody (clone RTK2758), (Cat No 400539), Biolegend. It is optimized for use in flow cytometric analysis. This antibody was chosen as an isotype control after screening on a variety of resting, activated, live, and fixed mouse, rat and human tissues. According to manufacturer's website each lot of this antibody is quality control tested by immunofluorescent staining with flow cytometric analysis as negative control. Use at concentrations comparable to those of the specific antibody of interest. Product citations:
1.Hou X, et al. 2020. Cell Reports. 28(1):172-189.e7.. PubMed
2.Monslow J, et al. 2020. Am J Pathol. 1118:190. PubMed
3. Go DM, et al. 2021. Cell Mol Gastroenterol Hepatol. 12:715. PubMed
Antibody profile in Antibody registry: https://www.antibodyregistry.org/AB_11126979

#PE Rat IgG2a, κ Isotype Ctrl Antibody (clone RTK2758), (Cat No 400508), Biolegend. It is optimized for use in flow cytometric analysis. This antibody was chosen as an isotype control after screening on a variety of resting, activated, live, and fixed mouse, rat and human tissues. According to manufacturer's website each lot of this antibody is quality control tested by immunofluorescent staining with flow cytometric analysis as negative control. Use at concentrations comparable to those of the specific antibody of interest. Product citations:
1. Combes F, et al. 2018. Neoplasia. 20:848. PubMed
2. Hou X, et al. 2020. Cell Reports. 28(1):172-189.e7.. PubMed
3. Li B, Schmidt N 2016. PLoS One. 11: 0162427. PubMed
Antibody profile in Antibody registry: https://www.antibodyregistry.org/AB_326530

#Annexin V Alexa fluor 647, (clone 640907), (Cat No A23204), Invitrogen. Annexin V staining to detect apoptotic cells can only be done on live cells and tissue.
1.Ito N, et al. 2007. J Leukoc Biol. PubMed ID: 16968820
2.Kubo S, et al. 2005. J Biol Chem. PubMed ID: 16020543
Antibody profile in Antibody registry: https://www.antibodyregistry.org/AB_2341149

#GR-1 PE-Cy7, (clone RB6-8C5), (Cat No 108415), Biolegend. The antibody has been validated for reactivity with mouse tissue or proteins. It is optimized for use in flow cytometric analysis of antibody surface-stained cells. According to manufacturer's website the suggested use of this ≤0.25 µg per million cells in 100 µl volume. Product Citations:
1.Katsumura KR, et al. 2018. Proc Natl Acad Sci U S A. 115:E10109. PubMed
2.Tran NT, et al. 2019. Cell Rep. 28:3510. PubMed
3.Lawson H, et al. 2021. Stem Cell Reports. 16:2784. PubMed
Antibody profile in Antibody registry: https://www.antibodyregistry.org/AB_313380

#GR-1 PerCP, (clone RB6-8C5), (Cat No 108425), Biolegend. The antibody has been validated for reactivity with mouse tissue or proteins. It is optimized for use in flow cytometric analysis of antibody surface-stained cells. According to manufacturer's website the suggested use of this ≤1.0 µg per million cells in 100 µl volume. Product Citations:
1.Jayachandran R, et al. 2019. Immunity. 50:152. PubMed
2. Man SM et al. 2016. Cell. 167(2):382-396 . PubMed
3.Han X, et al. 2017. Int J Mol Sci. 10.3390/ijms18050942. PubMed
Antibody profile in Antibody registry: https://www.antibodyregistry.org/AB_893560

#Ly6G PE-Cy7, (clone 1A8), (Cat No 127617), Biolegend. The antibody has been validated for reactivity with mouse tissue or proteins. It is optimized for use in flow cytometric analysis of antibody surface-stained cells. According to manufacturer's website the suggested use of this ≤ 0.25 µg per million cells in 100 µl volume. Product Citations:

1.Laura C Burzynski et al. 2019. Immunity. 50(4):1033-1042 . PubMed
2.Argüello RJ, et al. 2020. Cell Metab. 32:1063. PubMed
3.Bernasconi R, et al. 2021. EMBO Mol Med. :e14392. PubMed
Antibody profile in Antibody registry: https://www.antibodyregistry.org/AB_1877262

Ly6G APC, (clone 1A8), (Cat No 127613), Biolegend. The antibody has been validated for reactivity with mouse tissue or proteins. It is optimized for use in flow cytometric analysis of antibody surface-stained cells. It is recommended that the reagent be titrated for optimal performance for each application. According to manufacturer's website the suggested use of this ≤0.06 µg per million cells in 100 µl volume. Product Citations:
1.Patras KA, et al. 2019. J Innate Immun. :1. PubMed
2.Yu-Han Chang et al. 2017. Immunity. 47(5):943-958 . PubMed
3.Li A, et al. 2020. Oncol Lett. 1.899305556. PubMed
Antibody profile in Antibody registry: https://www.antibodyregistry.org/AB_1877163

Ly6C FITC, (clone AL-21), (Cat No 553104), BD Biosciences. The antibody has been validated for reactivity with mouse tissue or proteins. It is optimized for use in flow cytometric analysis of antibody surface-stained cells. It is recommended that the reagent be titrated for optimal performance for each application. Product Citations:
1.Cerwenka A et al, J Immunol. 1998; 161(1):97-105.
2.Jutila DB, et al. 1994 ; 41(1):49-57.
3.Jutila MA et al, Eur J Immunol. 1988.
Antibody profile in Antibody registry: https://www.antibodyregistry.org/AB_394628

#Siglec-F PE, (clone E50-2440), (1/50 dilution), (Cat No 562068), BD Biosciences. The antibody has been validated for reactivity with mouse tissue or proteins. It is optimized for use in flow cytometric analysis of antibody surface-stained cells. It is recommended that the reagent be titrated for optimal performance for each application. Product Citations:
1. Angata T et al, J Biol Chem. 2001; 276(48):45128-45136.
2.Crocker PR et al, Trends Immunol. 2001; 22(6):337-342.
Antibody profile in Antibody registry: https://www.antibodyregistry.org/AB_10896143

#Siglec-F BV421, (clone E50-2440), (Cat No 562681), BD Biosciences. The antibody has been validated for reactivity with mouse tissue or proteins. It is optimized for use in flow cytometric analysis of antibody surface-stained cells. It is recommended that the reagent be titrated for optimal performance for each application. Product Citations:
1. Angata T et al, J Biol Chem. 2001; 276(48):45128-45136.
2.Crocker PR et al, Trends Immunol. 2001; 22(6):337-342.
Antibody profile in Antibody registry: https://www.antibodyregistry.org/AB_2722581

#CD11c Percp.Cy5.5, (clone N418), (Cat no 117327), Biolegend. The antibody has been validated for reactivity with mouse tissue or proteins. It is optimized for use in flow cytometric analysis of antibody surface-stained cells. For flow cytometric staining, the suggested use of this reagent is ≤1.0 µg per million cells in 100 µl volume. It is recommended that the reagent be titrated for optimal performance for each application. Product Citations:
1. Kitagawa S, et al. 2022. NPJ Vaccines. 7:115. PubMed
2.Li X, et al. 2022. Nat Commun. 13:2794. PubMed
3.McNamara HA, et al. 2020. Cell Host Microbe. 572:28. PubMed
Antibody profile in Antibody registry: https://www.antibodyregistry.org/AB_2129642

#CD11b V500, (clone M1/70), (Cat No 562127), BD Biosciences. The antibody has been validated for reactivity with mouse tissue or proteins. It is optimized for use in flow cytometric analysis of antibody surface-stained cells. It is recommended that the reagent be titrated for optimal performance for each application. Product Citations:
1. Ault KA et al. J Immunol. 1981; 126(1):359-364.
2.Greimers R et al.Cytometry. 1996; 23(3):205-217.
3.Lagasse E et al. J Immunol Methods. 1996; 197(1-2):139-150.
4.Springer T et al. Eur J Immunol. 1978; 8(8):539-551.
5.Springer T et al. Eur J Immunol. 1979; 9(4):301-306.
Antibody profile in Antibody registry: https://www.antibodyregistry.org/AB_10893815

#CD11b BV421, (clone M1/70), (Cat No 562605), BD Biosciences. The antibody has been validated for reactivity with mouse tissue or proteins. It is optimized for use in flow cytometric analysis of antibody surface-stained cells. It is recommended that the reagent be titrated for optimal performance for each application. Product Citations:
1. Ault KA et al. J Immunol. 1981; 126(1):359-364.
2.Beller DI et al. J Exp Med. 1982; 156(4):1000-1009.
3.Driver DJ et al. J Immunol. 2001; 167(3):1393-1405.
4. Kaji Ket al. J Immunol. 2001;166(5):3256-3265.
Antibody profile in Antibody registry: https://www.antibodyregistry.org/AB_11152949

#MERTK FITC, (clone 2B10C42), (Cat No 151503), Biolegend. The antibody has been validated for reactivity with mouse tissue or proteins. It is optimized for use in flow cytometric analysis of antibody surface-stained cells. For flow cytometric staining, the suggested use of this reagent is ≤0.5 µg per million cells in 100 µl volume. It is recommended that the reagent be titrated for optimal performance for each application.
Product Citations:
1. Wang H, et al. 2022. Curr Protoc. 2:e446. PubMed
2.Zhu B, et al. 2021. Immunity. 54(6):1200-1218.e9. PubMed
3.Bagayoko S, et al. 2021. PLoS Pathog. 17:e1009927. PubMed
Antibody profile in Antibody registry: https://www.antibodyregistry.org/AB_2617034

#CD64 PE, (clone X54-5/7.1), (Cat No 139303), Biolegend. The antibody has been validated for reactivity with mouse tissue or proteins. It is optimized for use in flow cytometric analysis of antibody surface-stained cells. For flow cytometric staining, the

suggested use of this reagent is ≤1.0 µg per million cells in 100 µl volume. It is recommended that the reagent be titrated for optimal performance for each application. Product Citations:
1. Marchelletta RR, et al. 2021. J Clin Invest. 131:. PubMed
2.Afkhami S, et al. 2022. Cell. 185:896. PubMed
3.De Vries LCS, et al. 2021. J Crohns Colitis. 15:617. PubMed
Antibody profile in Antibody registry: https://www.antibodyregistry.org/AB_10613467

#MHCII FITC, (clone AF6-120.1), (Cat No 562011), BD Biosciences. The antibody has been validated for reactivity with mouse tissue or proteins. It is optimized for use in flow cytometric analysis of antibody surface-stained cells. It is recommended that the reagent be titrated for optimal performance for each application. Product Citations:
1. Beck BN et al. J Immunol. 1986; 136(8):2953-2961.
2.Cohn LE et al. Proc Natl Acad Sci U S A.1986; 83(3):747-751.
3.Hattori M, et al. Science. 1986; 231(4739):733-735.
4.Nabozny GH et al. J Exp Med. 1996; 183(1):27-37.
Antibody profile in Antibody registry: https://www.antibodyregistry.org/AB_10894585

#MHCII PE-CF594, (clone AF6-120.1), (Cat No 562824), BD Biosciences. The antibody has been validated for reactivity with mouse tissue or proteins. It is optimized for use in flow cytometric analysis of antibody surface-stained cells. It is recommended that the reagent be titrated for optimal performance for each application. Product Citations:
1. Beck BN et al. J Immunol. 1986; 136(8):2953-2961.
2.Cohn LE et al. Proc Natl Acad Sci U S A.1986; 83(3):747-751.
3.Hattori M et al. Science. 1986; 231(4739):733-735.
4.Nabozny GH et al. J Exp Med. 1996; 183(1):27-37.
5.Wall KA et al. J Immunol. 1983; 131(3):1056-1064.
Antibody profile in Antibody registry: https://www.antibodyregistry.org/AB_2737819

#CD45.1 FITC, (clone A20), (Cat No 110705), Biolegend. The antibody has been validated for reactivity with mouse tissue or proteins. It is optimized for use in flow cytometric analysis of antibody surface-stained cells. For flow cytometric staining, the suggested use of this reagent is ≤1.0 µg per million cells in 100 µl volume. It is recommended that the reagent be titrated for optimal performance for each application. Product Citations:
1. Lawson H, et al. 2021. Stem Cell Reports. 16:2784. PubMed
2.Vasanthakumar A, et al. 2020. Nature. 579:581. PubMed
3. Koyama M, et al. 2015. J Exp Med. 212: 1303 - 1321. PubMed
4.Guo H, Cooper S, Friedman A, et al. 2017. PLoS One. 10.1371/journal.pone.0150809. PubMed
Antibody profile in Antibody registry: https://www.antibodyregistry.org/AB_313494

#CD45.2 APC-Cy7, (clone 104), (Cat No 109823), Biolegend. The antibody has been validated for reactivity with mouse tissue or proteins. It is optimized for use in flow cytometric analysis of antibody surface-stained cells. For flow cytometric staining, the suggested use of this reagent is =1.0 µg per million cells in 100 µl volume. It is recommended that the reagent be titrated for optimal performance for each application. Product Citations:
1.Ajith A, et al. 2021. Front Immunol. 12:687715. PubMed
2.Yamazaki S, et al. 2022. Mucosal Immunol. :. PubMed
3.Chang D, et al. 2020. Immunity. 53(3):614-626.e4. PubMed
Antibody profile in Antibody registry: https://www.antibodyregistry.org/AB_830788

#CD206 APC, (clone C068C2), (Cat No 141707), Biolegend. The antibody has been validated for reactivity with mouse tissue or proteins. It is optimized for use in flow cytometric analysis of antibody surface-stained cells. For flow cytometric staining, the suggested use of this reagent is ≤0.5 µg per million cells in 100 µl volume. It is recommended that the reagent be titrated for optimal performance for each application. Product Citations:
1.Jiang Z, et al. 2021. J Clin Invest. 131: . PubMed
2.Kiepura A, et al. 2021. Int J Mol Sci. 22:. PubMed
3.Yokozeki Y, et al. 2021. Biomed Res Int. 2021:7988320. PubMed
Antibody profile in Antibody registry: https://www.antibodyregistry.org/AB_10896057

#Rat IgG2a, κ Isotype Ctrl Antibody APC, (clone RTK2758), (Cat No 400511), Biolegend. This antibody was chosen as an isotype control after screening on a variety of resting, activated, live, and fixed mouse, rat and human tissues. According to manufacturer's website each lot of this antibody is quality control tested by immunofluorescent staining with flow cytometric analysis as negative control. Use at concentrations comparable to those of the specific antibody of interest. It is recommended that the reagent be titrated for optimal performance for each application. Product Citations:
1.Zenke S, et al. 2022. Nat Commun. 13:6459. PubMed
2.Li B, Schmidt N 2016. PLoS One. 11: 0162427. PubMed
3.Choi JG, et al. 2020. Front Immunol. 11:598556. PubMed
Antibody profile in Antibody registry: https://www.antibodyregistry.org/AB_2814702

#CD40 Pe-Cy5, (clone 3/23), (Cat No 124617), Biolegend. The antibody has been validated for reactivity with mouse tissue or proteins. It is optimized for use in flow cytometric analysis of antibody surface-stained cells. For flow cytometric staining, the suggested use of this reagent is ≤ 0.25 µg per million cells in 100 µl volume. It is recommended that the reagent be titrated for optimal performance for each application. Product Citations:
1.Hasbold J, et al. 1994. Eur. J. Immunol. 24:1835.
2.Bourgeois C, et al. 2002. Science 297:2060.
Antibody profile in Antibody registry: https://www.antibodyregistry.org/AB_2075923

#Rat IgG2a, κ Isotype Ctrl Antibody PE-Cy5, (clone RTK2758), (Cat No 400509), Biolegend. This antibody was chosen as an isotype control after screening on a variety of resting, activated, live, and fixed mouse, rat and human tissues. According to manufacturer's website each lot of this antibody is quality control tested by immunofluorescent staining with flow cytometric analysis as negative control. Use at concentrations comparable to those of the specific antibody of interest. It is recommended that the reagent be

titrated for optimal performance for each application. Product Citations:
1.Klimovich B, et al. 2022. J Exp Clin Cancer Res. 41:80. PubMed
2.Ma P, et al. 2014. Diabetes. 63:3458. PubMed
3.Park JS, et al. 2022. Arthritis Res Ther. 24:254. PubMed
4.Zhu Y et al. 2017. The Journal of Neuroscience. 37(9):2362-2376 . PubMed
Antibody profile in Antibody registry: https://www.antibodyregistry.org/AB_326531

#7-AAD (Cat No 420403), Biolegend. 7-AAD Viability Staining Solution can be used as a viability probe for methods of nonviable cell exclusion. It has a high DNA binding constant and is efficiently excluded by intact cells. It is useful for DNA analysis and dead cell discrimination during flow cytometric analysis. According to the manufacturer's recommendations, for dead cell exclusion, the cell pellet should be resuspended in 0.5 mL of Cell Staining Buffer, and 5 µl of 7-AAD per million cells should be added. The analysis of the cells should be done as soon as possible after the incubation with the dye. Product citations:
1.Paterson AM, et al. 2011. J. Immunol. 187:1097. PubMed
2. Wingren M, et al. 2012. Arterioscler Thromb Vasc Biol. 32:2000. PubMed
3. Sandu SK, et al. 2012. PNAS 109:20047. PubMed

#Sytox (Cat No S34862), Thermofisher Scientific. The SYTOX™ Dead Cell Stains in the Sampler Kit have been optimized for use in cell concentrations ranging from 1x104 to 1x106 cells per ml. SYTOX™ Cell Stains have been validated in several cell types including: Jurkat, ReH, CHO, Kg1a, 3T3, HeLa, HEK (primary cells), Lysed whole blood. SYTOX™ Dead Cell Stains have been tested in several applications including: Apoptosis, Cell cycle, Cell proliferation, Immunophenotyping assays. Product citations: 1. Biotechnol Adv 28, 255 (2010); 2. Methods Mol Biol 521, 449 (2009); 3. Anal Chem 81, 5517 (2009); 4. J Biol Chem 284, 15496 (2009); 5. Inflamm Res 58, 210 (2009); 6. J Food Prot 71, 2168 (2008); 7. J Ind Microbiol Biotechnol 35, 1261 (2008); 8. Exp Hematol 36, 909 (2008); 9. J Leukoc Biol 83,456 (2008); 10. Nat Protoc 2, 2295 (2007); 11. Mutat Res 630, 78 (2007); 12. Appl Environ Microbiol 72, 7829 (2006); 13. Environ Technol 27, 909 (2006); 14. J Clin Endocrinol Metab 91, 4154 (2006).

#Biotin Rat Anti-Mouse CD31, (clone MEC13.3), (Cat No 553371), BD Biosciences. The antibody has been validated for reactivity with mouse tissue or proteins. It is optimized for use in flow cytometric analysis of antibody surface-stained cells. It is recommended that the reagent be titrated for optimal performance for each application. Product Citations:
Baldwin HS et al. Development. 1994; 120(9):2539-2953.
Christofidou-Solomidou M et al. J Immunol. 1997; 158(10):4872-4878.
DeLisser HM et al. Am J Pathol. 1997; 151(3):671-677.
DeLisser HM et al. Immunol Today. 1994; 15(10):490-495.
Famiglietti J et al. J Cell Biol. 1997; 138(6):1425-1435.
Antibody profile in Antibody registry: https://www.antibodyregistry.org/AB_394817

#Biotin Rat Anti-Mouse CD16/CD32, (clone 2.4G2) (Cat No 553143), BD Biosciences. The antibody has been validated for reactivity with mouse tissue or proteins. It is optimized for use in flow cytometric analysis of antibody surface-stained cells. It is recommended that the reagent be titrated for optimal performance for each application. Product Citations:
1.Araujo-Jorge T et al. Infect Immun. 1993; 61(11):4925-4928.
2.Benhamou M et al. J Immunol. 1990; 144(8):3071-3077.
3.Jensen WA et al. Biochem Soc Trans. 2001; 29(6):840-846.
Antibody profile in Antibody registry: https://www.antibodyregistry.org/AB_394658

Biotin Rat Anti-Mouse CD45, (clone 30-F11), (Cat No 553078), BD Biosciences. The antibody has been validated for reactivity with mouse tissue or proteins. It is optimized for use in flow cytometric analysis of antibody surface-stained cells. It is recommended that the reagent be titrated for optimal performance for each application. Product Citations:
1.Johnson P et al. In: Herzenberg LA, Weir DM, Herzenberg LA, Blackwell C , ed. Weir's Handbook of Experimental Immunology, Vol 2. Cambridge: Blackwell Science; 1997:62.1-62.16.
2.Lagasse E et al. Nat Med. 2000; 6(11):1212-1213.
3.Ledbetter JA et al. Immunol Rev. 1979; 47:63-90.
4.Thomas ML. Annu Rev Immunol. 1989; 7:339-369.
Antibody profile in Antibody registry: https://www.antibodyregistry.org/AB_394608

#c-Raf Antibody, (Cat No 9422), Cell Signaling Technology. It is optimized for use in western blotting at concentrations 1:1000. c-Raf Antibody detects endogenous levels of total c-Raf protein. Species Reactivity: Human, Mouse, Rat, Monkey. Product citations:
1. Avruch, J. et al. (1994) Trends Biochem Sci 19, 279-83.
2. Chong, H. et al. (2001) EMBO J 20, 3716-27.
3. King, A.J. et al. (1998) Nature 396, 180-3.
4. Fabian, J.R. et al. (1993) Mol Cell Biol 13, 7170-9.
5. Mason, C.S. et al. (1999) EMBO J 18, 2137-48.
6. Zimmermann, S. and Moelling, K. (1999) Science 286, 1741-4.
Antibody profile in Antibody registry: https://www.antibodyregistry.org/AB_390808

#Phospho-c-Raf (Ser338) (56A6) Rabbit mAb, (Cat No 9427), Cell Signaling Technology. It is optimized for use in western blotting at concentrations 1:1000. Phospho-c-Raf (Ser338) (56A6) Rabbit mAb detects endogenous levels of c-Raf only when phosphorylated at Ser338. Species Reactivity: Human, Mouse, Rat. Product citations:
1.Avruch, J. et al. (1994) Trends Biochem Sci 19, 279-83.
2.Chong, H. et al. (2001) EMBO J 20, 3716-27.
3.King, A.J. et al. (1998) Nature 396, 180-3.
4.Fabian, J.R. et al. (1993) Mol Cell Biol 13, 7170-9.
Antibody profile in Antibody registry: https://www.antibodyregistry.org/AB_2067317

#p44/42 MAPK (Erk1/2) (137F5) Rabbit mAb, (Cat No 4695), Cell Signaling Technology. It is optimized for use in western blotting at concentrations 1:1000. p44/42 MAP Kinase (137F5) Rabbit mAb detects endogenous levels of total p44/42 MAP kinase (Erk1/Erk2) protein. Species Reactivity: Human, Mouse, Rat, Hamster, Monkey, Mink, D. melanogaster, Zebrafish, Bovine, Dog, Pig, C. elegans. Product citations:

1.Baccarini, M. (2005) FEBS Lett 579, 3271-7.
2.Meloche, S. and Pouysségur, J. (2007) Oncogene 26, 3227-39.
3.Roberts, P.J. and Der, C.J. (2007) Oncogene 26, 3291-310.
Antibody profile in Antibody registry: https://www.antibodyregistry.org/AB_390779

#Phospho-p44/42 MAPK (Erk1/2) (Thr202/Tyr204) (D13.14.4E) XP® Rabbit mAb, (Cat No 4370), Cell Signaling Technology. It is optimized for use in western blotting at concentrations 1:2000.
Phospho-p44/42 MAPK (Erk1/2) (Thr202/Tyr204) (D13.14.4E) XP® Rabbit mAb detects endogenous levels of p44 and p42 MAP Kinase (Erk1 and Erk2) when dually phosphorylated at Thr202 and Tyr204 of Erk1 (Thr185 and Tyr187 of Erk2), and singly phosphorylated at Thr202. Species Reactivity: Human, Mouse, Rat, Hamster, Monkey, Mink, D. melanogaster, Zebrafish, Bovine, Dog, Pig, S. Cerevisiae.
Product citations:
1.Roux, P.P. and Blenis, J. (2004) Microbiol Mol Biol Rev 68, 320-44.
2.Baccarini, M. (2005) FEBS Lett 579, 3271-7.
3.Meloche, S. and Pouysségur, J. (2007) Oncogene 26, 3227-39.
Antibody profile in Antibody registry: https://www.antibodyregistry.org/AB_2315112

#GAPDH (14C10) Rabbit mAb, (Cat No 2118), Cell Signaling Technology. It is optimized for use in western blotting at concentrations 1:1000.
GAPDH (14C10) Rabbit mAb detects endogenous levels of total GAPDH protein. Species Reactivity: Human, Mouse, Rat, Monkey, Bovine, Pig. Product citations:
1.Barber, R.D. et al. (2005) Physiol. Genomics 21, 389-95.
2.Hara, M.R. and Snyder, S.H. (2006) Cell Mol. Neurobiol. 26, 527-38.
3.Zheng, L. et al. (2003) Cell 114, 255-66.
4.Bae, B.I. et al. (2006) Proc. Natl. Acad. Sci. USA 103, 3405-9.
5.Wang, Q. et al. (2005) FASEB J. 19, 869-71.
Antibody profile in Antibody registry: : https://www.antibodyregistry.org/AB_561053

#Anti-rabbit IgG, HRP-linked Antibody, (Cat No 7074S), Cell Signaling Technology. It is optimized for use in western blotting at concentrations 1:1000 - 1:3000.
Designed for use with rabbit polyclonal and monoclonal antibodies, this affinity purified goat anti-rabbit IgG (heavy and light chain) antibody is conjugated to horseradish peroxidase (HRP) for chemiluminescent detection. This product is thoroughly validated with CST primary antibodies and will work optimally with the CST western immunoblotting protocol, ensuring accurate and reproducible results. Product citations:
1.Barber, R.D. et al. (2005) Physiol. Genomics 21, 389-95.
2.Hara, M.R. and Snyder, S.H. (2006) Cell Mol. Neurobiol. 26, 527-38.
3.Zheng, L. et al. (2003) Cell 114, 255-66.
4.Bae, B.I. et al. (2006) Proc. Natl. Acad. Sci. USA 103, 3405-9.
5.Wang, Q. et al. (2005) FASEB J. 19, 869-71.
Antibody profile in Antibody registry: https://www.antibodyregistry.org/AB_2099233

#Mouse PLET-1 Antibody, (Cat No MAB6917), Clone 700738, R&D Systems. According to manufacturer the optimized usage recommendations are It is unconjugated Ab optimized for use in IgG1 Immunohistochemistry, Flow Cytometry, Immunohistochemistry. Recommended concentration in flow cytometry is µg/106 cells. Specificity: Detects mouse PLET-1 in direct ELISAs. Species Reactivity: Mouse.
Antibody profile in Antibody registry: https://www.antibodyregistry.org/AB_10891499

Animals and other research organisms

Policy information about studies involving animals; ARRIVE guidelines recommended for reporting animal research, and Sex and Gender in Research

Laboratory animals

C57BL/6 and B6.SJL-Ptprca mice expressing the CD45.1 alloantigen (Ly5.1 PTP) on circulating leukocytes with C57BL/6 genetic background were purchased from Charles River Laboratories. Ccr2-/- (B6.129P2-Ccr2tm1Mae) mice were generated as described in Kuziel WA, et al., 1997, and backcrossed to the C57BL/6 background. Plet1 transgenic mice were generated in cooperation with Taconic Biosciences with a tdTomato (tandem dimer tomato red) reporter gene tandemly expressed with a floxed Plet1 gene on C57BL/6 background to obtain constitutive knock-in of T2A-tdTomato in the Plet1 locus (Plet1 reporter) with optional conditional knock-out of the Plet1 gene together with the reporter gene. This line was further crossbred with B6.129P2(C)-Cx3cr1tm2.1(cre/ERT2)Jung mice (Jackson) for the generation of tamoxifen-responsive Cx3cr1+ (BMDM-specific) Plet1 conditional knockout mice (abbreviated Cx3cr1iCre-Plet1flx/flx) by tamoxifen (TXF) feeding. Tamoxifen was administered via chow (Taconic Biosciences, GmbH, 0.4 g/kg) prior to the start of the experiment.

The age of all mice used in the experiments reported in the study is 10-14 weeks.

Housing conditions for the mice are as follows:
- light/dark cycle of 4/10 hours
- temperature maintained at 22+/-2°C
- relative humidity at 55+/-10%

Wild animals

The study did not involve wild animals

Reporting on sex

All experiments were performed in mice regardless their sex and no sub analysis were done relying on sex discrimination.

Field-collected samples	No field-collected samples were used in this study
Ethics oversight	Animal experiments were approved by the regional authorities of the State of Hesse (Regierungspräsidium Giessen) and by the Institutional Ethics Committee at the IBioBA Institute (G36-2022).

Note that full information on the approval of the study protocol must also be provided in the manuscript.

Flow Cytometry

Plots

Confirm that:

☒ The axis labels state the marker and fluorochrome used (e.g. CD4-FITC).

☒ The axis scales are clearly visible. Include numbers along axes only for bottom left plot of group (a 'group' is an analysis of identical markers).

☒ All plots are contour plots with outliers or pseudocolor plots.

☒ A numerical value for number of cells or percentage (with statistics) is provided.

Methodology

Sample preparation

Preparation of bronchoalveolar lavage for flow cytometry:
Mice were sacrificed by cervical dislocation and the trachea was exposed to insert a 21-gauge cannula via a small incision. Mice were then lavaged with 10x 500µl PBS/ 2mM EDTA. Bronchoalveolar lavage fluid (BALF) was stored on ice until further processing. Cells in BALF were pelleted by centrifugation at 1400rpm for 10min at 4°C and resuspended in FACS buffer (PBS-/-, 10 % FBS, 0.1 % NaN3). These cells were pelleted and incubated with mouse Fc-blocking reagent for 5 mins at 4°C and cells were stained with fluorochrome-conjugated antibodies for 30 min at 4 °C.
Annexin V staining, cells were resuspended in Annexin V staining buffer (10 mM HEPES, 140 mM NaCl, and 2.5 mM CaCl2).

Preparation of lung homogenates for flow cytometry:
Mice were sacrificed, lungs were perfused with sterile HBSS (Gibco) via right heart ventricle puncture. The lungs are filled slowly with 800-1500µL of dispase using a 21-gauge cannula via a small incision into trachea and tied with a suture thread to avoid the leakage of dispase. The lungs were then removed, after carefully dissecting out the heart and incubated for 40 min at room temperature in dispase. After removal of the trachea and proximal bronchial tree, the lungs were homogenized (GentleMACS, MACS Miltenyi Biotech) in DMEM/2.5 % HEPES with 0.01 % DNase (Serva) and filtered through 100 µm and 40 µm nylon filters. Obtained cells were pelleted by centrifugation at 500 g for 10 min at 4°C, re-suspended in 1ml DMEM and HEPES, counted, stained with the fluorochrome-labeled antibodies for 30 min at 4°C and stored on ice for flow cytometry analysis.

Isolation of primary murine alveolar epithelial cells (AEC):
After the lung homogenates wer prepared, cell suspensions were incubated with biotinylated rat anti-mouse CD45, CD16/32 and CD31 mAbs for 30 min at 37°C followed by incubation with biotin-binding magnetic beads and magnetic separation to deplete leukocytes and endothelial cells prior to further culture. AEC suspensions with a purity ≥ 90 % as determined by FACS were seeded in 24-well plates at a density of 250,000 cells/cm2, and cultured in DMEM enriched with HEPES, L-Glutamine, FCS, and pen/strep. After reaching the confluence cells were detached from the well using Accutase, and thereafter stained with the fluorochrome-labeled antibodies (proliferation assay, Ki67).

Instrument	BD LSRFortessa flow cytometer and BD AriaIII (BD Biosciences)
Software	FACS Diva v7.0 and FlowJo v10.7.0
Cell population abundance	Purities of sorted BMDM1/2 and isolated primary AEC were assessed by FACS and showed a purity ≥ 90% in all samples.
Gating strategy	The starting cell population was gated according to FCS/SSC followed by doublet exclusion gate. This was followed by dead cell exclusion (gate set according to unstained control). Usually, population gates were set according to the FMO control including an isotype control wherever possible.

☒ Tick this box to confirm that a figure exemplifying the gating strategy is provided in the Supplementary Information.
